# The Impact of Chemotherapy on the Ovaries: Molecular Aspects and the Prevention of Ovarian Damage

**DOI:** 10.3390/ijms20215342

**Published:** 2019-10-27

**Authors:** Charlotte Sonigo, Isabelle Beau, Nadine Binart, Michaël Grynberg

**Affiliations:** 1 Department of Reproductive Medicine and Fertility Preservation, Hôpital Antoine Béclère, 92140 Clamart, France; 2 Inserm U1185 Université Paris-Sud, Université Paris Saclay, 94276 Le Kremlin Bicêtre, France; isabelle.beau@u-psud.fr (I.B.); nadine.binart@inserm.fr (N.B.); 3 Inserm U1133 Université Paris Diderot, 75013 Paris, France

**Keywords:** fertility preservation, ovaries, burnout effect

## Abstract

Cancer treatment, such as chemotherapy, induces early ovarian follicular depletion and subsequent infertility. In order to protect gametes from the gonadotoxic effects of chemotherapy, several fertility preservation techniques—such as oocyte or embryo cryopreservation with or without ovarian stimulation, or cryopreservation of the ovarian cortex—should be considered. However, these methods may be difficult to perform, and the future use of cryopreserved germ cells remains uncertain. Therefore, improving the methods currently available and developing new strategies to preserve fertility represent major challenges in the area of oncofertility. Animal and ovarian culture models have been used to decipher the effects of different cytotoxic agents on ovarian function and several theories regarding chemotherapy gonadotoxicity have been raised. For example, cytotoxic agents might (i) have a direct detrimental effect on the DNA of primordial follicles constituting the ovarian reserve and induce apoptosis; (ii) induce a massive growth of dormant follicles, which are then destroyed; or (ii) induce vascular ovarian damage. Thanks to improvements in the understanding of the mechanisms involved, a large number of studies have been carried out to develop molecules limiting the negative impact of chemotherapy on the ovaries.

## 1. Introduction

In the past few decades, the significant diagnostic and therapeutic progress made in the field of oncology has improved the survival rates of children and young adults. However, it is now clearly established that these excellent results are achieved through treatments that have potentially deleterious impacts on reproductive function. In order to protect gametes from the gonadotoxic effects of chemotherapy and/or radiotherapy, several fertility preservation (FP) techniques, such as oocyte or embryo cryopreservation with or without ovarian stimulation or cryopreservation of the ovarian cortex, should be proposed [1]. However, the application of these methods may be limited by age, pubertal status, disease, and emergency. In addition, these procedures may be difficult to perform, and the future use of cryopreserved germ cells remains uncertain. Therefore, improving the FP methods currently available and developing new FP strategies represent major challenges in oncofertility.

Chemotherapy exerts toxicity on the ovaries directly. It is important to distinguish between the short- and long-term effects of drugs on the ovaries. Soon after the beginning of treatment, chemotherapy induces apoptosis of growing follicles, leading to temporary amenorrhea. The impact of drugs on fertility after healing concerns the effects of chemotherapy on the primordial follicular reserve as these treatments may lead to a premature loss and, at worst, primary ovarian insufficiency (POI). POI is a well-known long-term side effect of cancer chemotherapy treatment. The extent of ovarian damage depends upon several factors, of which the most important are the type of drug, its dosage, and the protocol [2]. Chemotherapeutic agents can be divided into five categories: alkylating agents, antitumor antibiotics, platinum-based drugs, antimetabolites, and taxanes. The mechanisms implicated in the gonadotoxicity of these molecules have been explored in various experimental models, such as analysis of histological female ovary sections after chemotherapy, animal models treated with injections, xenograft models, or cell cultures in the presence of active metabolites of chemical agents, and are not yet fully understood [3]. Several hypotheses have been proposed and could coexist. On the one hand, chemotherapeutic agents could exert direct toxicity on primordial follicles, inducing DNA damage and subsequent apoptosis. On the other hand, it has been suggested that these drugs could trigger an indirect depletion of primordial follicles by over-recruitment. Increasing knowledge of the possible mechanisms implicated in chemotherapy-induced ovarian damage will facilitate the development of new therapies, called fertoprotective agents [4], aimed at protecting the follicular reserve [5].

## 2. Follicular Ovarian Reserve and Its Regulation

In mammals, the follicular ovarian reserve, constituted by primordial follicles, is established early on in life then keeps declining regularly throughout the reproductive period. The pool of primordial follicles serves as a source of growing follicles and fertilizable eggs for the entire female reproductive life. Actually, each primordial follicle can remain quiescent for years; be activated and enter the growing process; or undergo atresia directly from the dormant stage [6]. To produce mature oocytes, activated primordial follicles develop through primary and secondary stages before acquiring an antral cavity. At the antral stage, most follicles undergo apoptotic degeneration and only a few of them grow further to reach the preovulatory stage under the cyclic gonadotropin stimulation that occurs after puberty [7].

The maintenance of female reproductive function implies the presence of a vast majority of quiescent primordial follicles and continuous repression of primordial follicle activation into early growing follicles. This activation, starting during fetal life, is finely controlled though maintaining a balance between inhibitory and stimulatory factors. In vitro experiments and genetically modified mouse or sheep models have enabled the decoding of the molecular mechanisms that control follicular activation. Numerous factors, such as growth factors, hormones, transcription factors, or cytokines, produced by oocytes and/or granulosa cells, can act in an autocrine, paracrine, or endocrine manner [8]. The quiescence of primordial follicles is maintained by several molecules including phosphatase and tensin homolog deleted on chromosome 10 (Pten), tuberous sclerosis complexes 1–2 (Tsc1–Tsc2) complex, Forkhead box protein O3A (Foxo3A), p27, anti-Müllerian hormone (AMH), and Forkhead box L2 (FoxL2) [6]. Many studies have highlighted the crucial roles of the phosphatidyl-inositol-3-kinase (PI3K) signaling pathway in oocytes in controlling follicular activation [9]. Indeed, in genetically modified mouse models, it was observed that the PI3K–Akt–mammalian target of rapamycin (mTOR) signaling pathway is crucial for the control of survival and activation of primordial follicles [10]. For example, Pten and Tsc1–2 are negative regulators of this signaling pathway and, in mice, the deletion of these genes from oocytes leads to primordial follicle activation and subsequent early follicular depletion. In these models, mTOR activity is accelerated within the oocyte, highlighting the critical role of this serine/threonine kinase in primordial follicle activation. The transcription factor FoxO3A, mainly expressed in the oocytes of resting follicles, acts downstream of the PI3K signaling pathway and appears to be the main actor involved in follicular activation [8]. At the same time, the survival of primordial follicles is maintained by other mechanisms involving PDK1 signaling, rpS6. Several studies have suggested the involvement of the autophagy process in the regulation of the ovarian reserve of primordial follicles. For example, autophagy is implicated in maintaining the primordial oocyte pool in murine newborns [11], and the induction of autophagy at birth seems to be a crucial step to preserve the stock of primordial follicles [12,13].

In the same manner, primordial follicle survival or apoptosis results from a balance between the expression of survival (antiapoptotic) and proapoptotic factors. Among these factors, the proteins B-cell lymphoma 2 (Bcl-2) and Bcl-2-associated X protein (BAX) likely play a critical role.

Thus, a synergistic and coordinated suppression of follicular activation, provided by multiple inhibitory and activator molecules, is necessary to preserve the primordial follicular stockpile in association with the process maintaining dormancy. Any disorder in these complex mechanisms can lead to a premature loss of the follicular reserve [14].

## 3. Follicular Atresia and Apoptosis

### 3.1. Pathophysiology: DNA Alteration, Follicular Atresia, and Apoptosis

The molecules used in chemotherapy induce alterations in the DNA. Double-stranded breaks (DSB) are one of the main DNA lesions caused by these cytotoxic agents and the most severe. DSB can, in turn, lead to either DNA repair pathways allowing cell survival or cell death by apoptosis [15]. DNA repair pathways differ according to the type of chemotherapeutic agent and may involve, for example, pATM, RAD51, or PARP1 proteins [15]. When the repair pathways are not sufficiently activated, DNA damage induces cellular apoptosis. This mechanism is mainly mediated by p63 protein (and, more specifically, the TAp63 isoform), which activates Bcl2-associated X (BAX) protein and the Bcl-2 antagonist killer (BAK) protein. BAX/BAK activation can be transmitted by TAp73 or secondary to p53 up-modulator of apoptosis) and phorbol-12-myristate-13-acetate–induced protein 1 activation [16]. These mechanisms are particularly complex within the ovaries and differ according to the type of chemotherapy molecule. A recent and extensive review discusses the induction and repair of DNA damage in the ovaries [15].

The impact of chemotherapeutic agents on growing follicles is well known, and apoptotic pathways have been well documented [5,17]. Almost all classes of drugs induce DNA alteration of granulosa cells and/or oocytes, leading to either apoptosis of growing follicles or the survival of mutagenic oocytes. This phenomenon often induces temporary amenorrhea [18]. More rarely, if fertilization occurs during drugs’ exposure, it can lead to spontaneous abortion or congenital abnormalities in the offspring [19]. These complications are closely related to the timing of oocyte exposure to cytotoxic treatment. Fertilization months or years after the end of protocol seems to be safe for offspring as these pregnancies are achieved from oocytes exposed in a dormant state, which remained genetically undamaged [20].

While apoptosis and atresia in growing follicles in response to chemotherapeutic agents have been well investigated, the nature of these mechanisms in dormant follicles is still under debate [15]. According to several studies, the main chemotherapeutic agents induce follicular depletion by directly affecting the primordial follicles entering massively into atresia [15]. Overall, rodent models as well as models of human ovarian xenograft or in vitro ovary cultures were used to investigate the impact of chemotherapy on primordial follicles. Cyclophosphamide is a widely used alkylating agent and is recognized as one of the most gonadotoxic drugs. It has been shown to induce DNA double-stranded breaks and activate the DNA damage response in a human ovarian xenograft model [21]. These results were confirmed in in vitro ovarian cultures with cyclophosphamide active metabolite [22,23,24] or after in vivo cyclophosphamide injection [25]. In the same manner, in vitro analysis of newborn mouse ovaries revealed DNA damage and apoptosis of primordial follicles after cisplatin treatment [26,27], which were further confirmed after in vivo injection in newborn or adult mice [16,25]. Recently, a model of a xenograft of human cortex ovaries in nude mice revealed the same results [28]. Furthermore, similar effects with similar models were also found following doxorubicin exposure [29,30].

**Figure 1:**
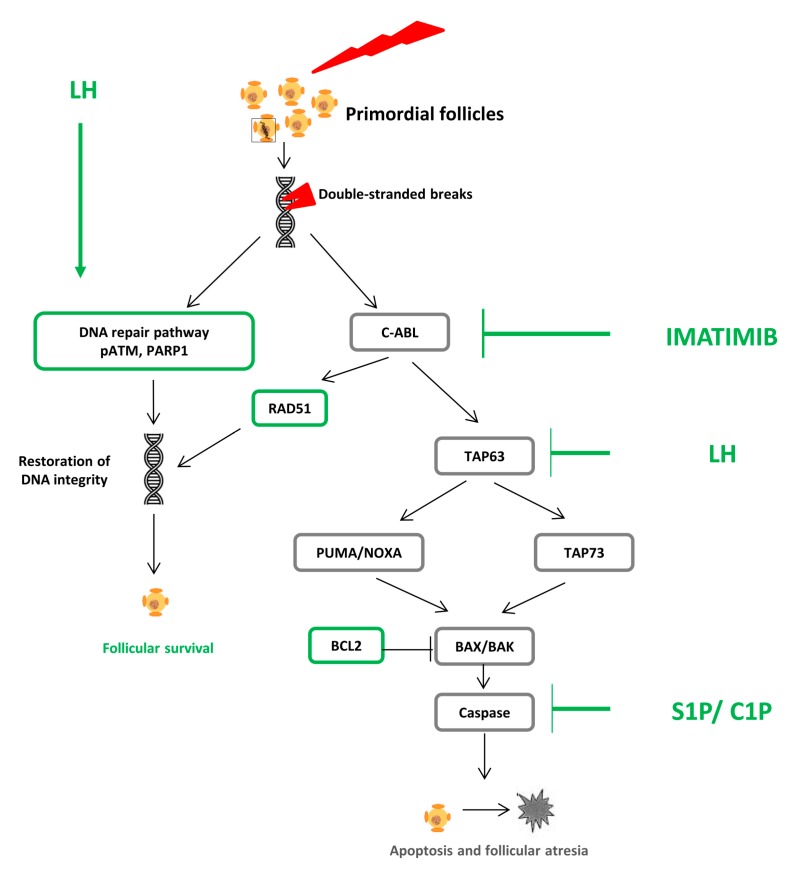
First hypothesis of chemotherapy-induced ovarian damage: apoptosis of primordial follicles. Chemotherapy induces double-stranded DNA breaks in the oocyte. If not repaired, they induce follicular atresia by apoptosis. Several molecules (in green), acting mainly on the different stages of the apoptotic pathway, have been proposed to avoid follicular atresia and maintain the pool of reserve follicles.

The improvement of knowledge of the specific apoptotic and DNA repair pathways involved in the ovarian damage induced by chemotherapy will reveal targets for protective agents to reduce or prevent ovarian damage (Figure 1) [3].

### 3.2. Fertoprotective Agents

#### 3.2.1. Sphingosine 1 Phosphate and Ceramide 1 Phosphate

Sphingosine-1-phoshate (S1P) is a membrane sphingolipid involved in several physiological processes, including apoptosis of ovarian follicles. Indeed, it was shown that the sphingomyelin pathway regulates the developmental death of oocytes and S1P protects the ovarian reserve from radiation injuries [31]. Later, in an animal model, S1P injection directly into the ovaries was shown to decrease the apoptosis of primordial follicles induced by chemotherapy and thus protect fertility [32,33]. In a human ovarian xenograft model, S1P can block the human apoptotic follicle death induced by cyclophosphamide and doxorubicin and preserve the primordial follicle stockpile [34,35]. Moreover, S1P seems to reduce the atresia of primordial follicles that occurs during the slow freezing and thawing of human ovarian cortical strips, confirming its protective role [36]. Recently, ceramide 1 phosphate (C1P), another sphingolipid, was also found to be a potential ovarian protective agent as ovarian administration of this drug reduces the ovarian damage induced by cyclophosphamide and protects the ovarian reserve via the inhibition of apoptosis and improvement of stromal vasculature [37]. However, one study had conflicting results, demonstrating that S1P was not effective against apoptosis in rats after intraperitoneal cyclophosphamide treatment [38].

One of the major limitations of these treatments is that S1P and C1P must be administered by continuous administration or injection directly into the ovaries. Nevertheless, recently, a long-acting oral form of an S1P analog has been developed and its impact on the ovaries was evaluated in a rat model [39]. It was suggested that this treatment might decrease spontaneous follicular apoptosis, making these molecules potentially appropriate for human use.

However, even if S1P might be a promising fertility preservation strategy in the future, additional studies have to be conducted to confirm the protective role of this molecule, to evaluate possible interference with chemotherapy, and to evaluate the impact of this strategy on offspring.

#### 3.2.2. Imatinib

Imatinib is a competitive tyrosine kinase inhibitor and, more specifically, a c-Abl kinase inhibitor. This protein is implicated in the apoptotic pathway induced by DNA damage in activating TAP63 transcriptional activity. Clinically, it is used for the treatment of hemopathies or other cancers. Based on its role as a c-Abl kinase inhibitor, imatinib was evaluated as a molecule to prevent the primordial follicle loss caused by cisplatin as this drug was shown to induce DNA damage and subsequent apoptosis in primordial follicles via TAP63 activation. It was hypothesized that imatinib could prevent the TAP63 accumulation and activation induced by cisplatin and thus impede follicle apoptosis. This molecule was first evaluated in 2009 by Gonfloni et al. in a mouse model [26]. In this study, the authors observed the occurrence of massive primordial and primary follicle depletion in cisplatin-treated mice, whereas they noted a significant rescue of these follicles in the ovaries of mice simultaneously treated with cisplatin and imatinib. Furthermore, they showed that this treatment had a long-term impact on fertility and reproductive outcomes. Similar results were found by the same team in 2012 [40], while others have confirmed these results using in vitro newborn ovary cultures [41] and in vitro culture and subrenal grafting of mouse ovaries [42]. Nevertheless, two studies have also contested these results, finding that imatinib did not protect primordial follicles from cisplatin-induced apoptosis and did not prevent impaired fertility [28,43].

Thus, due to the existence of conflicting results, additional studies are needed to evaluate whether imatinib could be a new treatment to limit cisplatin gonadotoxicity. Moreover, as imatinib interferes with the apoptotic pathway, it will be crucial to show that imatinib does not interfere with the antitumor activity of cisplatin.

#### 3.2.3. Molecules Interfering with the DNA Repair Pathway

Following spontaneously occurring, or chemotherapy-induced, DNA damage, the efficiency of the DNA repair pathway is a critical determinant of a cell’s survival. Thus, several studies have tried to develop molecules aiming to induce DNA repair instead of the apoptosis pathway to encourage follicle survival and limit follicular depletion.

For example, Rad 51 is a protein implicated in DNA repair after double-strand breaks. It was shown that, in an in vitro oocyte culture model, DNA damage in oocytes can be induced by doxorubicin and that oocytes possess the machinery and capability for repairing such DNA damage through Rad51 activation [44]. So, strategies manipulating Rad51 could be potential candidates to limit follicle depletion due to chemotherapy.

Recently, Rossi et al. reported the protective effect of luteinizing hormone (LH) on the primordial follicle pool of prepubertal mouse ovaries against cisplatin-induced follicular depletion [27]. First, these authors conducted an in vitro analysis and showed that LH treatment of prepubertal ovarian fragments generated antiapoptotic signals, reducing the oocyte level of proapoptotic TAp63 protein and favoring the DNA repair pathway in the oocytes. Thereafter, they showed that the administration of a single dose of LH to prepubertal female mice, concomitantly with cisplatin injection, inhibited the depletion of the primordial follicle reserve caused by the drug. If this protective role of recombinant LH is confirmed, it could be a very interesting candidate as this molecule is already available to women. Thus, clinical studies could be conducted relatively quickly.

## 4. Follicular Activation

### 4.1. Physiopathology of Ovarian Reserve Depletion Due to Follicular Activation

A more recent theory suggests that chemotherapy, such as cyclophosphamide or cisplatin, induces follicular depletion through the massive growth of resting follicles, occurring simultaneously with the apoptosis of growing follicles [45]. Recruitment of primordial follicles would be secondary to the activation of the PI3K signaling pathway, whose role in follicle quiescence has been well-established by many knockout mouse models as well as in vitro studies on human ovarian cortex fragments [8,46]. In addition, cytotoxic agents destroy growing follicles, resulting in a reduction in AMH secretion. As this hormone is supposed to inhibit primordial follicles’ recruitment, its decrease amplifies follicular activation and the subsequent depletion of the follicular reserve. In the first study revealing this hypothesis, no primordial follicles showed signs of apoptosis [45]. Other studies, using the same mouse model, confirmed this hypothesis, called the “burnout effect” [47,48]. Thus, the burnout effect consists of the trigger of recruitment of dormant follicle growth, mediated by an upregulation in the PI3K/PTEN/Akt pathway, occurring simultaneously with large follicle apoptosis and resulting in a reduction of AMH secretion. The route by which chemotherapy induces the activation of this signaling pathway remains unclear. It may be via the direct influence on the oocytes and pregranulosa cells of primordial follicles [49]. This theory was also supported when using other cytotoxic agents such as cisplatin [50,51]. In consideration of this theory, Lande et al. showed that, in vitro, phosphoramide mustard, a cyclophosphamide metabolite, enhances human primordial follicle activation in developing follicles [52]. This theory could also explain the alteration of the follicular reserve induced by the presence of an ovarian endometrioma [53] or massive follicular loss secondary to ovarian cortex transplantation [54,55]. Nevertheless, the molecular mechanism by which chemotherapy activates the PI3K pathway within primordial follicles is not known.

**Figure 2:**
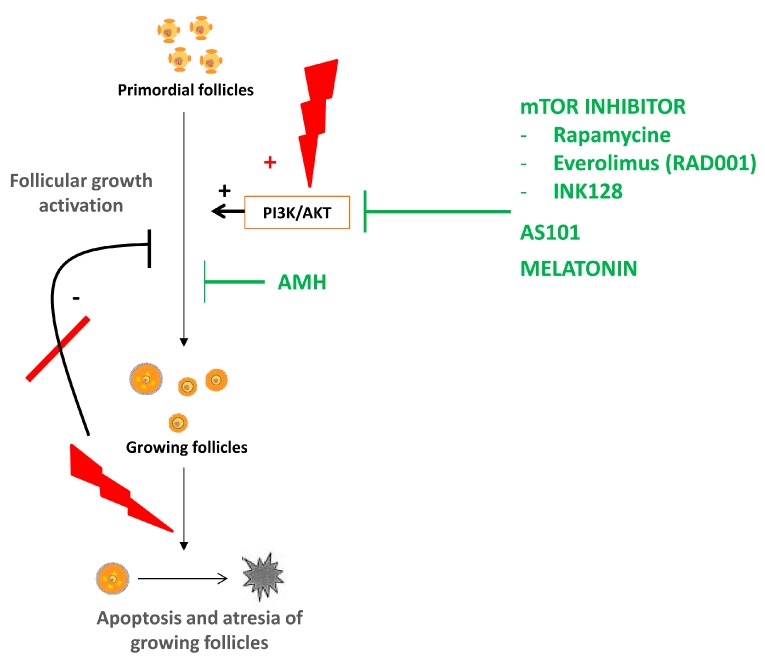
Second hypothesis of chemotherapy-induced ovarian damage: “burnout” effect. Chemotherapy induces both activation of the PI3K pathway and atresia of growing follicles. These two actions cause follicular depletion by massive activation of the primordial follicles. Molecules that interfere with the PI3K pathway have been developed to block the accelerated recruitment of primordial follicles (in green). (+: activates, - inhibits)

In accordance with this hypothesis, in the last few years, several investigations have been carried out to develop new molecules that would preserve the ovarian reserve by inhibiting the PI3K pathway and follicular activation (Figure 2) [3,56].

### 4.2. Fertoprotective Agents

#### 4.2.1. AS101

AS101 [ammonium trichloro(dioxoethylene-o,o′)tellurate] is a nontoxic immunomodulatory compound that modulates the PI3K–Pten–Akt pathway [57]. It has been shown to reduce the negative hematologic and dermatologic side effects of chemotherapy [58]. As cyclophosphamide was found to activate the PI3K pathway, inducing primordial follicle recruitment and subsequent follicular depletion of ovarian reserve, AS101 was investigated as a treatment to prevent cyclophosphamide-induced follicle loss in a mouse model [45]. Ultimately, in vivo treatment of mice with AS101 was found to reduce the cyclophosphamide-induced depletion of primordial follicles. No increase in fetal malformation was observed in mice previously treated with AS101, indicating the safety of this treatment for offspring. This treatment was the first one tested and yielded encouraging results; nevertheless, to date, no other studies using this molecule to prevent cyclophosphamide ovarian damage have been performed.

#### 4.2.2. Anti-Müllerian Hormone

AMH is a glycoprotein hormone expressed by the granulosa cells surrounding the oocytes. It is produced by follicles from the primary stage of development until selection for dominance, and plays a key role during folliculogenesis. As it has been shown to limit the activation of primordial follicles in in vivo or in vitro mouse models [59,60,61], it was suggested in three recent studies that this hormone could be an effective treatment option to limit chemotherapy-induced gonadotoxicity [48,62,63].

Kano et al. reported that, in mice, superphysiologic doses of AMH delivered either by a recombinant protein via osmotic pumps or gene therapy could limit the primordial follicle loss induced by cyclophosphamide, doxorubicin, or cisplatin [62]. The protective effects of AMH vary between drugs, suggesting that different mechanisms for ovarian damage are induced by different chemotherapeutic agents.

Recently, we assessed the protective effect of AMH in pubertal mice treated with cyclophosphamide [48]. In this model, we showed that the ovaries of cyclophosphamide-treated mice were depleted of primordial follicles, whereas the number of primordial and early-growing follicles was similar to that in controls among the ovaries of mice treated with concomitant injections of cyclophosphamide and AMH. Then, we showed that 15 weeks after the end of the treatment, the number of ovulated eggs after ovarian stimulation was significantly reduced in cyclophosphamide-treated mice and rescued by AMH co-administration. The molecular mechanisms underlying these effects were explored. Interestingly, an investigation of the PI3K signaling pathway showed that the phosphorylation of FoxO3A was significantly lower in mouse ovaries treated with AMH. This transcription factor, expressed in the nucleus of primordial follicles, plays an essential role in the maintenance of primordial follicles in a quiescent state [6]. The phosphorylation of FoxO3A induces the protein nuclear export, leading to the activation of primordial follicles [8]. Our results suggested that AMH might inhibit primordial follicle recruitment by preventing cytoplasmic shuttling of FoxO3A induced by cyclophosphamide. Moreover, in this study, we also provide evidence of a possible role of autophagy in the preservation of the follicular pool reserve. Indeed, we showed that AMH administration was able to induce autophagy in ovaries and a possible mechanism to explain the modulation of AMH-induced autophagy might implicate FOXO3A as this factor was shown to be related to autophagy activation [64]. These results are in accordance with those of other studies suggesting the involvement of autophagy in the regulation of follicular ovarian reserve [13,65].

Further, Roness et al. confirmed the fertoprotective role of recombinant AMH in the same mouse model as pharmacological administration of AMH during chemotherapy treatment reduced follicle activation and primordial follicle loss and significantly improved reproductive outcomes [63]. Interestingly, they also showed that AMH does not interfere with the therapeutic actions of chemotherapy.

These data indicate that AMH represents a potential novel treatment for limiting the primordial follicle depletion induced by chemotherapy. Nevertheless, these promising results need to be confirmed further. As AMH is produced only by the ovaries and acts through a specific receptor mainly expressed by the ovaries, this hormone might be of particular interest since it could act as a targeted therapy without interfering with physiological mechanisms or the efficacy of chemotherapy.

#### 4.2.3. Melatonin

Primarily revealed as a secretory product of the pineal gland, melatonin (N-acetyl-5-methoxytryptamine) is commonly used in various biological processes such as treating insomnia. Moreover, melatonin can be used as a potential therapeutic adjuvant during chemotherapy as it has been shown to reduce some adverse effects of drugs [66]. Interestingly, melatonin is also produced in various tissues including reproductive tissues such as the ovaries and the placenta [67], and melatonin receptors are present in the oocytes and granulosa cells of various species, including humans [68,69]. Some studies have revealed that melatonin treatment could limit the depletion of germ cells in the gonads during chemotherapy. In rats, melatonin administration prevents cisplatin-induced testicular toxicity and reduces sperm motility [70]. Recently, it was suggested as a new fertoprotective agent option against the ovarian damage induced by chemotherapy [51,56,71].

Jang et al. evaluated the protective effect of melatonin on cisplatin-treated ovaries in a mouse model [71]. They demonstrated that combined treatment with melatonin and cisplatin significantly prevented primordial follicle loss in cisplatin-treated ovaries. The molecular mechanisms implicated were also analyzed. In accordance with the burnout theory, these authors showed that the protection effect of melatonin was mediated by suppressing follicular recruitment through activation of the PI3K–Akt–FoxO3a signaling pathway. Nevertheless, this protective effect was partial. In addition, the same authors recently confirmed these results and revealed that ghrelin enhances the protective effect of melatonin against cisplatin-induced ovarian failure in a mouse model [51]. The molecular mechanisms implicated were evaluated and revealed that the coadministration of ghrelin and melatonin inhibited cisplatin-induced phosphorylation of Pten and FoxO3A. As FoxO3A phosphorylation induces its cytoplasmic translocation and subsequent follicular activation, the inhibition of this process maintains the primordial follicles in a dormant state.

This treatment seems promising, but knowledge of the details of the molecular mechanism of melatonin’s protective response against chemotherapy-induced ovarian damage and the need for evaluation of this impact on ovaries requires further studies.

#### 4.2.4. mTOR Inhibitors

mTOR is a serine/threonine kinase implicated in several crucial processes such as cell growth, proliferation, autophagy, and survival [72]. In mice, accelerated mTOR activity in the oocyte activates the primordial follicles, resulting in POI [73]. mTOR stimulators increase the activation of primordial follicles in animal models and mTOR inhibitors block the primordial-to-primary follicle transition [74]. According to these data, and after having confirmed the burnout theory, recent studies used, in a mouse model, mTOR inhibitors to preserve the ovarian reserve from cyclophosphamide-induced follicular depletion [75,76,77]. Goldman et al. explored the use of the clinically approved drug everolimus (RAD001) or the inhibition of mTORC1/2 with the experimental drug INK128, showing that mTOR inhibition preserves the ovarian reserve, as measured through primordial follicle counts and serum AMH levels [75]. Moreover, cyclophosphamide-treated mice had significantly fewer offspring, whereas cotreatment with mTOR inhibitors preserved normal fertility. The protective effect of everolimus was also demonstrated against cisplatin-induced gonadotoxicity in an in vivo mouse model [77]. As everolimus can be used in the treatment of some breast cancers, this approach represents a very interesting option for fertility preservation during conventional chemotherapy. On the other hand, Zhou et al. observed that cotreatment of chemotherapy with rapamycin, another mTOR inhibitor, prevented the follicle growth wave caused by cyclophosphamide treatment and significantly reduced primordial follicle loss [76]. Rapamycin is an inhibitor of the mTOR pathway, shown previously to inhibit the accelerated activation of primordial follicles of *Pten*−/− rat ovaries [78].

## 5. Vascular Damage

### 5.1. Physiopathology

Alterations in the ovarian stroma and vascularization are another mechanism potentially implicated in chemotherapy-induced follicle loss [3,18]. Indeed, vascular damage, revealed by decreased ovarian blood flow and reduction in ovarian size, has been demonstrated in women [79] and in mice following doxorubicin administration [80]. In addition, the histological analysis of human ovaries previously exposed to chemotherapy revealed that a thickening and hyalinization of cortical stromal blood vessels had occurred in association with the disorganization of blood vessels in the ovarian cortex and cortical fibrosis [81].

### 5.2. Fertoprotective Agents

#### G-CSF

In light of the vascular damage induced by chemotherapy, granulocyte colony-stimulating factor (G-CSF) was tested as a fertoprotective agent. Subsequently, it was determined that treatment with G-CSF decreased chemotherapy-induced ovarian follicle loss and extended the time to premature ovarian insufficiency in female mice treated with cyclophosphamide and busulfan [82]. Later, similar protective effects were found, as follicle counts and serum AMH levels were significantly increased in mice treated with cisplatin and G-CSF as compared with mice treated with cisplatin alone [83].

## 6. Other Molecules as Candidate Fertoprotective Agents

### 6.1. GnRH Analogs

Tested in 1995 in rhesus monkeys, gonadotropin-releasing hormone (GnRH) analogs were the first agents considered as possible chemoprotective molecules against cyclophosphamide ovarian damage [84]. Subsequent studies evaluated the possible protective effects in a rodent model with contradictory results [85,86,87,88,89,90]. In a more recent study, it was proven that ovarian damage occurred even in the absence of FSH, suggesting that the inhibition of the pituitary–gonadal axis is not involved in ovarian protection during GnRH agonist treatment [90]. Nevertheless, other mechanisms potentially implicated in this protective influence were suggested to be vascular effects or the upregulation of antiapoptotic molecules [91,92,93].

Several clinical studies have been performed to assess, in women, the ability of GnRH analogs to protect ovaries from chemotherapy ovarian damage. POI incidence, chemotherapy-induced amenorrhea, menses recovery, or pregnancy rates were evaluated in cancer patients who received GnRH analogs or not at the time of chemotherapy treatment. Conflicting results were reported [92,94]. Elsewhere, a meta-analysis of randomized clinical trials revealed diverse conclusions about the ability of GnRH analogs to preserve fertility [95,96,97,98]. Nevertheless, while clinical evidence for the efficacy of this treatment is still being debated, the safety of this strategy has already been clearly demonstrated. Thus, this treatment could be proposed for all young women requiring chemotherapy, although gamete cryopreservation should be performed, if possible, for women who want to preserve their fertility.

### 6.2. Tamoxifen

Tamoxifen is an estrogen receptor antagonist and is currently used as an adjuvant therapy for hormone-sensitive breast cancer. In a rodent model, the administration of tamoxifen significantly decreased doxorubicin- or cyclophosphamide-induced follicle loss [99]. Similar results were obtained in cultured rat ovaries [100]. Nevertheless, the molecular mechanisms of this protective effect during chemotherapy have not been discovered yet.

### 6.3. Other Molecules

In the past few years, several other molecules have been observed to decrease chemotherapy-induced ovarian damage in the drive to preserve fertility, including Chinese herbal medicine [101], fennel [102], sildenafil citrate [103], tocotrienol [104], genistein [105], and erythropoietin [106].

## 7. Conclusions

Improving the knowledge of the molecular mechanisms involved in chemotherapy-induced ovarian damage can lead to the development of treatments to limit follicular depletion in vivo [3,18,56]. The molecular mechanisms implicated in the protective role of these different agents are more or less clear. Table 1 summarizes the main fertoprotective agents that have been evaluated in a mouse model, their mechanism(s) of action, and the proposed mechanism(s) to explain ovarian protection.

Although histological studies of human ovaries were carried out years ago to assess the impact of the disease and treatments on the gonads [107,108], this type of research is more difficult to perform today due to ethical concerns. Nowadays, the assessment of the gonadotoxicity of chemotherapy is often based on organotypic or cell culture models in vitro. In vivo studies in rodents as well as models of human ovarian xenograft are also commonly used to investigate the impact of chemotherapy on primordial follicles and the potential protective role of fertoprotective agents. Moreover, the main chemotherapies used in these fundamental studies were cisplatin, cyclophosphamide, or doxorubicin. A recent review has been published that critically discusses the damaging effects of the most common chemotherapeutic compounds (cyclophosphamide, cisplatin, and doxorubicin) on the ovaries [109]. In clinical practice, the protocols applied incorporate a combination of several drugs. Therefore, the results obtained should be extrapolated to women, but caution should be used when interpreting the clinical relevance of such findings. Indeed, it is difficult to mimic the doses and protocols used, and ovarian physiology and responses to treatments can differ.

For these molecules to be used in clinical practice and studied in women, it is essential that they do not interfere with the therapeutic action of chemotherapy or important physiological processes. However, as apoptosis represents the main mechanism of anticancer action, apoptosis inhibitors could reduce the anticancer effect of chemotherapy. In addition, by blocking the death of oocytes with DNA alterations, some molecules could facilitate the survival of damaged germ cells and thus promote infertility, an increased risk of spontaneous miscarriages, or fetal malformations. Finally, the PI3K pathway is a ubiquitous pathway and the molecules modulating this pathway could interfere with various physiological processes.

New therapies aimed at limiting follicular loss and protecting the ovaries would be of great interest. They could be used in combination with the currently available fertility preservation techniques and administered regardless of age, pathology, or proposed treatment. Moreover, they would also prevent against hormonal deficiencies and their consequences (e.g., pubertal delay, osteoporosis). Finally, these treatments may be of particular interest to women with altered ovarian reserve parameters, in whom no fertility preservation method can be proposed.

## Figures and Tables

**Table 1:** Main molecules evaluated in an in vivo rodent model to limit chemotherapy-induced follicular depletion.

Fertoprotective Mechanism	Fertoprotective Agent		References
Inhibition of primordial follicular apoptosis	Sphingosine 1 phosphate ceramide 1 phosphate	Membrane sphingolipid	[32,33,34,35,37,38]
	Imatinib	Competitive tyrosine-kinase inhibitor (c-Abl kinase inhibitor)	[16,26,40,42]
	GNF2	c-Abl kinase inhibitor	[40]
	LH	Gonadotrophine	[27]
Inhibition of primordial follicle recruitment	AS101	PI3K modulator	[45]
	Melatonin	Pineal hormone	[51,71]
	Rapamycin	mTOR inhibitor	[76]
	Everolimus (and INK128)	mTORC1/mTORC2 inhibitor	[75,77]
	AMH	Ovarian hormone	[48,62,63]
Several mechanisms proposed (e.g., vascular effect, follicular recruitment inhibition)	GnRH analogs	Inhibition of the pituitary-gonadal axis	[85,86,88,89,90]
Vascular effect	G-CSF	Granulocyte colony-stimulating factor	[82,83]
Prevention of chemotherapy nuclear activation	Bortezomib	Proteasome inhibitor	[30]
